# Patients’ motivations for participating in cardiovascular clinical trials: a local perspective

**Published:** 2009-08

**Authors:** LJ Burgess, NU Sulzer, F Hoosain, N Leverton, S Bliganut, S Emanuel

**Affiliations:** Department of Cardiology/TREAD Research, Stellenbosch University/Tygerberg Hospital, Parow, Western Cape, South Africa; Department of Cardiology/TREAD Research, Stellenbosch University/Tygerberg Hospital, Parow, Western Cape, South Africa; Department of Cardiology/TREAD Research, Stellenbosch University/Tygerberg Hospital, Parow, Western Cape, South Africa; Department of Cardiology/TREAD Research, Stellenbosch University/Tygerberg Hospital, Parow, Western Cape, South Africa; Department of Cardiology/TREAD Research, Stellenbosch University/Tygerberg Hospital, Parow, Western Cape, South Africa; Department of Cardiology/TREAD Research, Stellenbosch University/Tygerberg Hospital, Parow, Western Cape, South Africa

## Abstract

**Objective:**

To investigate patients’ motivations for participating in cardiovascular clinical trials.

**Methods:**

Patients attending TREAD Research, located within Tygerberg Hospital, Parow, Western Cape, between January 2005 and May 2006 were approached to participate in the study. Consenting patients were given a validated questionnaire to complete in their home language. All questionnaires were anonymous and 250 consecutive patients completed the questionnaire. They provided basic demographic data and rated their response to 18 statements concerning factors that may or may not have influenced their decision to participate in a clinical trial.

**Results:**

The mean (± SD) age of subjects was 56.3 ± 10.9 years. A large percentage of the respondents were unemployed (66.5%). Access to medical care was a motivation for the majority of patients (90.5%). Ninety-six per cent of patients appreciated the regular follow up they received as trial participants; 90% of patients entered the trial to receive medication, which they could otherwise not afford. A substantial 98% of patients participated to learn more about their disease. Almost all (99%) wanted to further the scientific understanding of their condition. A reassuring 94% of subjects felt that they were not pressurised into the study; 80% of patients disagreed that participation in clinical trials was an easy way to obtain money.

**Conclusions:**

Access to medical care and making a contribution to scientific knowledge are two of the most common motivations for participation in cardiovascular clinical trials. The role of remuneration is relatively unimportant.

## Summary

The international drug development industry is under constant pressure to reduce the cost and duration of clinical trials. Countries such as South Africa, South America and India are becoming attractive options for sponsors.^1^ The South African advantage lies in its ability and reputation to produce high-quality data at 20 to 30% less than the cost in the United States.^1^ In addition, the disparate socio-economic conditions found in this country result in access to patients with diseases of both the industrialised and developing worlds.^1^

This growing trend to conduct clinical research in developing nations has led to a concern regarding the apparent exploitation of vulnerable subjects by international sponsor companies. Subjects who may be considered vulnerable are those individuals more likely to be unduly influenced in their decision-making process, for example regarding matters of healthcare.^2^ These could be participants whose economic status may make them more susceptible to financial incentives.^2^

Little, if any, attention has been paid to the actual motivations behind subjects participating in clinical trials in South Africa. Understanding the reasons behind patients enrolling in clinical trials will assist in the development of patient recruitment strategies and improve retention of subjects. The aim of this study was to explore why patients enrol in clinical trials, specifically trials investigating atherosclerosis and other chronic diseases of lifestyle.

## Methods

This study was conducted by TREAD Research, a private clinical trial research unit located within Tygerberg Hospital, Parow, Western Cape. This unit focuses on trials investigating chronic diseases of lifestyle and is affiliated to the Cardiology Unit of Stellenbosch University.

Patients attending the research unit between January 2005 and May 2006 were approached to participate in the study. Subjects were given an informed consent form to read and sign if they wished to participate. The study was approved by the Committee for Clinical Trials, Stellenbosch University. Consenting patients were given a validated questionnaire to complete in their home language. All questionnaires were anonymous and patients were instructed to place their completed questionnaire in a sealed box in the research unit’s waiting room. Patients were assigned a random consecutive number and no identifying data were recorded on the questionnaire.

A total of 250 consecutive patients approached over this time period completed the questionnaire. Patients were asked to provide basic demographic data, which included their gender, age, home language, current occupation and monthly earnings. They were then asked to rate their response to 18 statements concerning factors that may or may not have influenced their decision to participate in a clinical trial. Patients were able to choose between ‘strongly agree’, ‘agree’, ‘partly agree’ or ‘disagree’.

## Statistical analysis

All questionnaires were examined for missing information. Any questions that had not been answered were not included in the final analysis. Data were entered into a spreadsheet by an independent data capturer. All data were analysed using Statistica Software Version 7.0 (StatSoft©, Inc, USA) on a personal computer. Parametric data are presented as mean ± standard deviation (SD). The monthly income data are expressed as the median (lower and upper quartiles). Categorical data are presented as percentages of the total number of respondents for that particular question.

## Results

The mean (± SD) age of subjects was 56.3 ± 10.9 years. The majority of the respondents were Afrikaans speaking (71.7% vs 27.9% English). There were 118 male subjects (47.0%) and 132 female subjects (52.6%). Only 4.4% of the respondents were not currently participating in a clinical trial. A large percentage of the respondents were unemployed (66.5%), with only 31.5% registering a paying job or business. The median (lower and upper quartile) monthly family income was R1 800.00 (R740.00 and R5 200). All respondents (100%) indicated they had given voluntary informed consent to participate in a clinical trial prior to any procedures being performed.

**Fig. 1.**
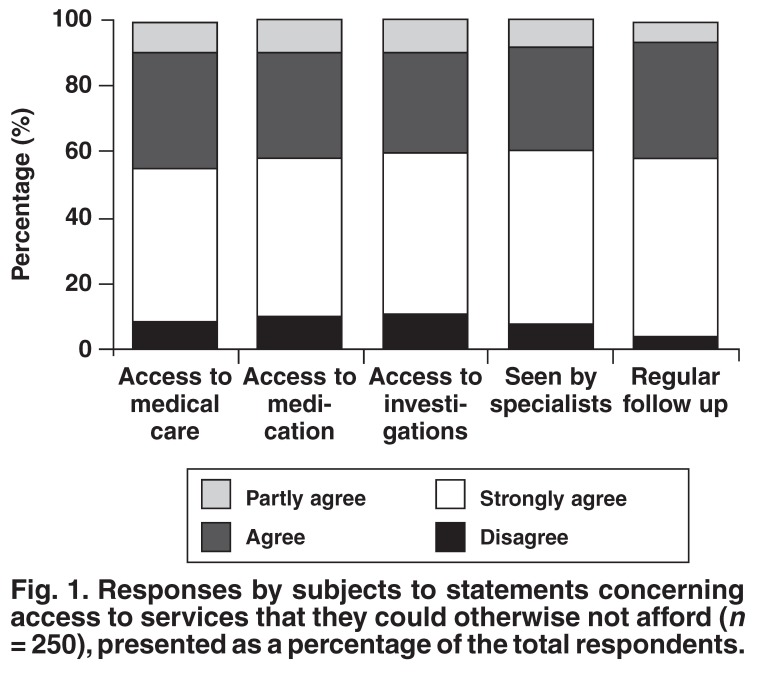
Responses by subjects to statements concerning access to services that they could otherwise not afford (*n* = 250), presented as a percentage of the total respondents.

**Fig. 2.**
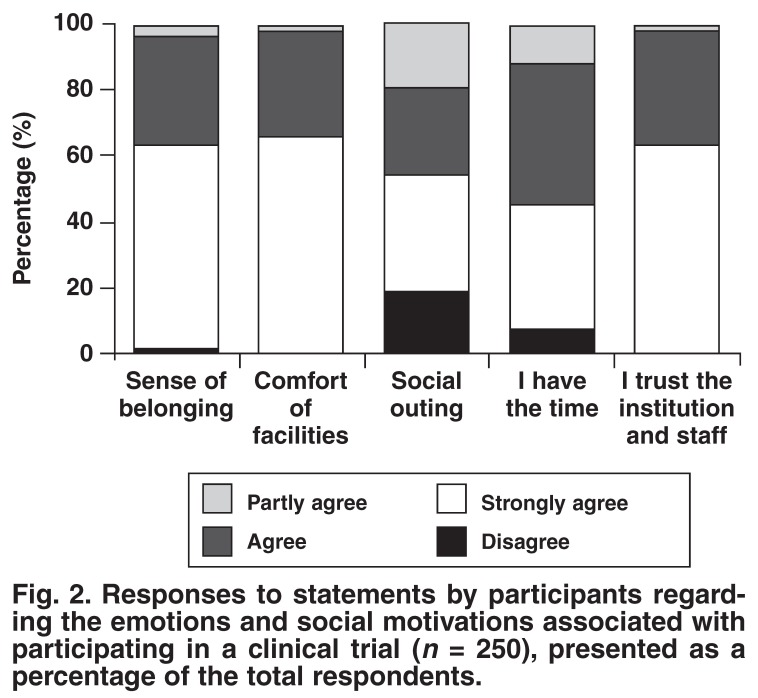
Responses to statements by participants regarding the emotions and social motivations associated with participating in a clinical trial (*n* = 250), presented as a percentage of the total respondents.

**Fig. 3.**
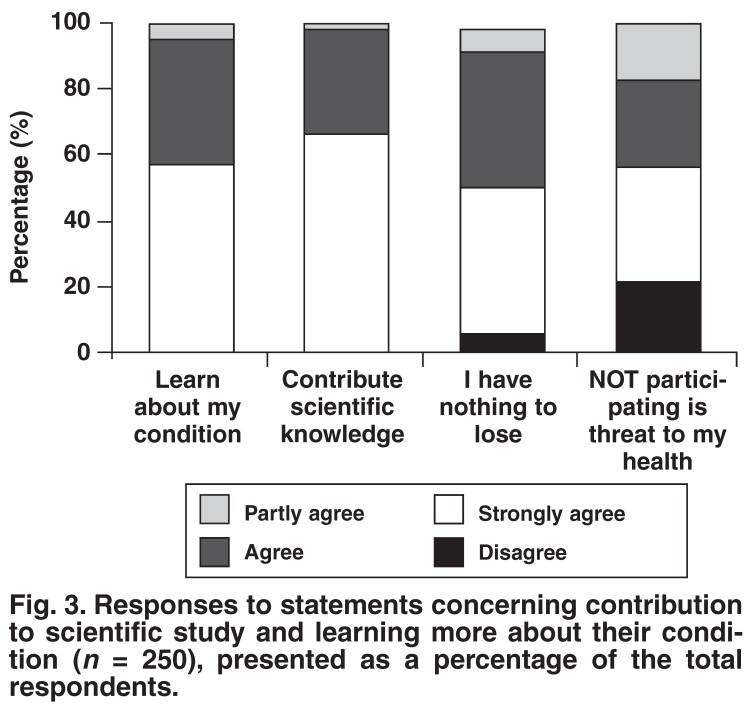
Responses to statements concerning contribution to scientific study and learning more about their condition (*n* = 250), presented as a percentage of the total respondents.

**Fig. 4.**
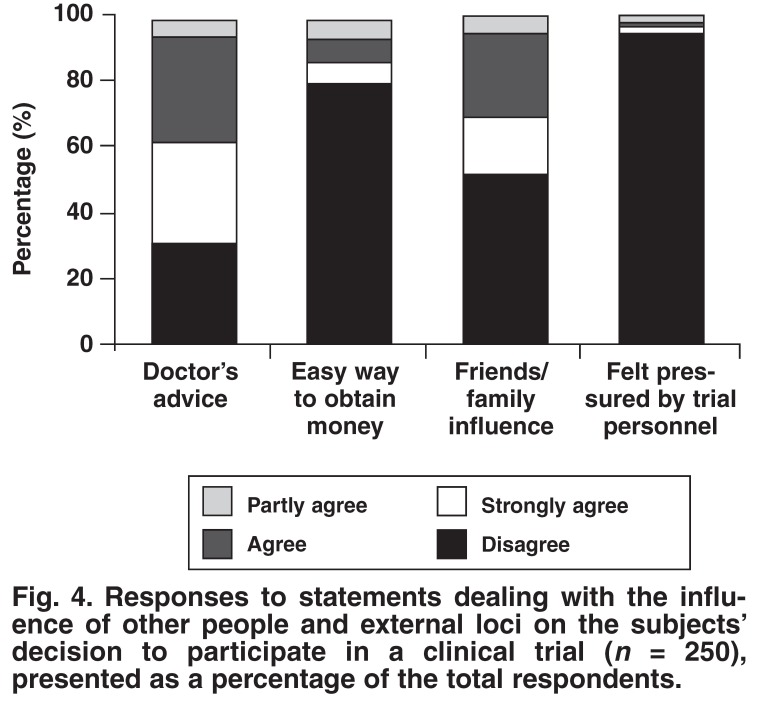
Responses to statements dealing with the influence of other people and external loci on the subjects’ decision to participate in a clinical trial (*n* = 250), presented as a percentage of the total respondents.

The subjects’ responses to the 18 statements regarding factors influencing their decision to participate in a clinical trial are presented below in Figs 1, 2, 3, 4. Subjects could choose to ‘strongly agree’, ‘agree’, ‘partly agree’ or ‘disagree’ with any particular statement. The responses are presented in groups, according to statement themes, for ease of reference.

Fig. 1 presents the responses to statements concerning access to heath services that the subject could otherwise not afford. Fig. 2 presents the responses to statements regarding the emotions and social motivations concerned with participating in a clinical trial. Fig. 3 presents the motivations for contributing to scientific study and further understanding their condition. Finally, Fig. 4 presents responses to statements regarding the influence of other people and external loci (including money) to the subjects’ decision to participate in a clinical trial.

## Discussion

Access to quality medical care and medication is a human right but often due to the pressure on government healthcare sector resources, many state patients are forced to wait in lengthy queues or receive sub-optimal treatment. As trial subjects, patients have access to high-quality medical care, including often expensive investigations, that they could otherwise not afford. Access to these services was a motivation for the majority of patients (Fig. 1).

Regular follow up forms part of most clinical trial protocols, whereas the experience in state healthcare facilities is somewhat different. It is not therefore surprising that 96% of the patients appreciated the regular follow up they received as trial participants. Access to healthcare and regular follow up was also cited by British diabetes trial patients as a motivation for participation in clinical trials, emphasising that this is not a motivation reserved solely for vulnerable populations.^3^

Access to medication, despite the possibilities of receiving a placebo or the existence of unknown side effects, was identified as a contributory factor to patient involvement in clinical research (Fig. 1). It is not a rare occurrence for patients to be testing drugs that are not yet available on the South African pharmaceutical market but are marketed in North American or Western Europe. Despite the possible risks, 90% of the patients reported that they enter into trials to receive medication that they could otherwise not afford.

There appear to be emotional and social motivations for participating in clinical research. This is supported by the large percentage of patients who agreed that they felt a sense of belonging at the clinic (Fig. 2). The value of individual contact and being treated with respect is therefore apparent. The chatter in the waiting room is itself proof of the fact that clinic visits are viewed as a social outing (Fig. 2). Eighty-one per cent of patients appreciated the opportunity of meeting other patients with similar problems and sharing their experiences. Almost all patients agreed that they enjoyed the comfort and quality of the facilities and that they trusted the institution and staff involved in the trials, an indication of the importance of the trial staff ’s relationship with patients enrolled in trials.

Clinical trials are often time consuming due to the length of the consultations and the time it takes to complete all protocol-related tests. This time factor plays a major role in the patient recruitment process and needs to be discussed during the informed consent process. Only 7% of patients at this trial centre felt that they did not have the time required to participate in a clinical trial.

One of the greatest benefits of participating in a clinical trial is the understanding patients gain of their disease and the management thereof. A substantial 98% of patients agreed that their involvement in the clinical research project was related to learning more about their disease (Fig. 3). The contribution to further the scientific understanding of their condition is an altruistic motivation expressed by 99% of the subjects. This motivation is shared by trial participants in developed countries who include contributing to science as one of the main motivations for wanting to participate in a clinical trial.^4^,^5^

It was intriguing that 78% of patients felt that not participating in a trial posed a serious threat to their health. This may indicate a level of patient vulnerability in state patients who do not receive adequate healthcare, and enrol in trials to safeguard their health. This is not a motivation found only in developing countries, however, as this sentiment was shared by the group of British diabetic patients who listed ‘reducing the threat of my disease’ as one of their strongest motivations for participating in a clinical trial.^3^

The influence of other people and external loci on deciding whether to participate in a clinical trial was also investigated (Fig. 4). The influence of a doctor in the decision-making process is often debated, especially in vulnerable subjects who are frequently uneducated and easily impressed (known as the ‘whitecoat syndrome’). It is reassuring to see that while 67% of patients were advised by their doctor to take part, 94% of subjects felt that they were not pressurised into the study by trial personnel.

The influence of family and friends should not be underestimated, as was discovered in this study. Almost half of all patients claimed that they were influenced by their family and friends to participate in a clinical trial. It is evident that the families of patients play an important role in patient recruitment in this country.

The influence of money proved to be, in the patients’ opinion, of minor importance (Fig. 4). Eighty per cent of patients disagreed that participation in clinical trials was an easy way to obtain money. Remuneration appeared to play a very small contributory role in patient recruitment. This is supported by the response given by patients enrolling in an HIV vaccine-efficacy trial, where only 14% listed financial reimbursement as a reason for enrolling in the trial.^5^

The potential limitations of a self-administered questionnaire should be taken into account when analysing the results of such a study. The authors did validate the questionnaire prior to implementation in order to minimise any possible misunderstanding or misinterpretation.

## Conclusion

This study presents, from a patient’s perspective, the reasons behind some South African patients enrolling in clinical trials. There are few, if any, such data currently available. Although there seem to be numerous factors that influence a patient’s decision to participate in a clinical trial, access to medical care and making a contribution to scientific knowledge appear to be two of the most common motivations. The role of remuneration, despite this being a comparatively poor and vulnerable population, was relatively unimportant.

These results not only provide an understanding of trial patients’ motivations, but also supply vital information to use in patient recruitment and retention. In addition, these results suggest that potentially vulnerable South African subjects are not being exploited by large foreign pharmaceuticals but rather that this is a mutually beneficial relationship. Every clinical trial needs to be conducted with the patient in mind if it is to be successful.^6^ These results offer an insight into the factors playing a role in patients’ decision making and therefore are invaluable to clinical researchers.
